# Analytical performance of reagent for assaying tau protein in human plasma and feasibility study screening neurodegenerative diseases

**DOI:** 10.1038/s41598-017-09009-3

**Published:** 2017-08-24

**Authors:** Shieh-Yueh Yang, Ming-Jang Chiu, Ta-Fu Chen, Chin-Hsien Lin, Jiann-Shing Jeng, Sung-Chun Tang, Yen-Fu Lee, Che-Chuan Yang, Bing-Hsien Liu, Hsin-Hsien Chen, Chau-Chung Wu

**Affiliations:** 1 MagQu Co., Ltd., Xindian District, New Taipei City, 231 Taiwan; 2 Department of Neurology, National Taiwan University Hospital, College of Medicine, National Taiwan University, Taipei, 100 Taiwan; 3 Graduate Institute of Brain and Mind Sciences, College of Medicine, National Taiwan University, Taipei, 100 Taiwan; 4 Department of Psychology, National Taiwan University, Taipei, 100 Taiwan; 5 Graduate Institute of Biomedical Engineering and Bioinformatics, National Taiwan University, Taipei, 116 Taiwan; 6 Departments of Internal Medicine and Primary Care Medicine, College of Medicine, National Taiwan University, Taipei, 100 Taiwan

## Abstract

Immunomagnetic reduction (IMR), which involves the use of antibody-functionalized magnetic nanoparticles to specifically label target biomarkers, was utilized to develop an assay for total tau protein in human plasma. The analytic properties of the IMR assay on tau protein were investigated. The limit of detection was found to be 0.026 pg/ml. Other properties such as Hook effect, assay linearity, dilution recovery range, reagent stability, interference test, and spiked recovery were also characterized. The ultra-sensitive IMR assay was applied to detect the plasma tau protein levels of subjects with prevalent neurodegenerative diseases, such as Alzheimer’s disease (AD), mild cognitive impairment (MCI) due to AD, Parkinson’s disease (PD), frontotemporal dementia (FTD) and vascular dementia (VD). The concentrations of plasma tau protein in patients with VD, PD, MCI due to AD, FTD, and AD patients were higher than that of healthy controls. Using an ROC curve analysis, the cutoff value for discriminating dementia patients from healthy controls was 17.43 pg/ml, resulting in 0.856 and 0.727 for clinical sensitivity and specificity, respectively. The area under the ROC curve was 0.908. These results imply that the IMR plasma tau assay would be useful to screen for prevalent neurodegenerative diseases.

## Introduction

Tau protein is abundant in neurons in the central nervous system^1–3^. Its main function is to stabilize axonal microtubules^4–6^. When neurons become fibrillary, tau protein is released abundantly in the brain, leading to a change in the tau protein concentration in cerebrospinal fluid (CSF). Neurofibrillary tangles are frequently observed in brain biopsies of subjects with neurodegenerative diseases (NDD). Thus, patients with NDD show abnormal levels of tau protein in their CSF. In recent decades, several groups have reported an elevation of CSF tau protein in Alzheimer’s disease (AD) patients^7–10^. The results showed that synaptic/axonal degeneration or loss is one of the pathologies for AD. The elevation of CSF tau protein levels was also found in patients with frontotemporal dementia (FTD). Furthermore, independent studies have revealed that concentrations of CSF tau protein in FTD are between those of AD and healthy controls^11–13^. This result implies that the neurofibrillary tangle formation occurs in FTD patients. In the 2010s, the exploration of tau protein in CSF was not limited to AD and FTD but was initiated for patients suffering from Parkinson disease (PD) or dementia with Lewy bodies (DLB). However, changes in CSF tau protein concentrations of PD or DLB have not been consistent^14–21^. For example, studies conducted by Parnetti *et al*. and Arai *et al*. concluded that patients with DLB show higher concentrations of CSF tau protein than that of healthy controls^8, 9^. However, Kanemaru *et al*. and Andersson *et al*. indicated that there is no significant difference in CSF tau protein concentration between DLB and healthy controls^16, 17^. However, many reports have revealed that patients with Parkinson’s disease dementia (PDD) have lower levels of tau protein in CSF than healthy controls^18–21^. Nevertheless, Přikrylová *et al*. found an increase in the CSF tau protein concentration for PD and DLB^22^. The inconsistent results among studies could be attributed to the truly tiny changes in the concentrations of tau protein in CSF in Parkinson’s disease. A more precise assay technology might be needed to clearly discriminate the CSF tau protein concentrations between patients with PD/DLB and healthy controls.

With the development of ultrahigh-sensitivity assay technologies, such as modified enzyme-linked immunosorbent assay^23^, multiplex electrochemiluminescence^24, 25^, surface-based fluorescence intensity distribution analysis^26^, multiplexed flowmetrix analysis^27, 28^, selected reaction-monitoring mass spectrometry^29^, and single-molecule array (SIMOA)^30, 31^, the precision of tau protein detection has improved. Furthermore, the investigation of tau protein in dementia is not limited to CSF but has expanded to include human plasma^32, 33^. For example, Zetterberg *et al*. applied SIMOA to quantitatively detect total tau protein in plasma for normal controls (n = 25) and patients with AD (n = 54) or MCI (n = 75)^33^. The study revealed that the plasma tau protein concentration of AD patients, (8.80 ± 10.1) pg/ml, is relatively higher than that of MCI patients, (4.68 ± 4.25) pg/ml, and normal controls, (4.43 ± 2.83) pg/ml. These ultrahigh-sensitivity assay technologies have created a trend of exploring tau protein levels in human plasma in dementia.

Some authors of the present study developed an ultrahigh-sensitivity assay technology called immunomagnetic reduction (IMR)^34–36^. Chiu *et al*. applied IMR to assay plasma tau protein for 107 normal controls, 24 patients with MCI due to AD, and 31 patients with early-stage AD, in Taiwan. Normal controls showed levels of (16.16 ± 9.09) pg/ml for plasma tau protein, whereas patients with MCI due to AD showed levels at (33.33 ± 7.77) pg/ml^37^. A clear difference in the concentration of plasma tau protein was found between controls and patients with MCI due to AD. Moreover, Chiu *et al*. noted that early-stage AD patients show much higher concentrations of plasma tau protein, (53.57 ± 22.87) pg/ml. The continuous increase in plasma tau protein concentrations from normal controls to MCI and to early-stage AD is found to result from the atrophy of the hippocampus. Lue *et al*. recruited 16 normal controls and 16 AD patients at the Banner Sun Health Institute to have their plasma tau protein concentrations analyzed by IMR. A higher level of plasma tau protein was observed for AD patients compared with normal controls^38^. These results reveal the high correlation between the concentration of plasma tau protein and the clinical diagnoses and the feasibility of precisely assaying tau protein in human plasma by utilizing IMR.

In IMR, magnetic nanoparticles functionalized with antibodies and well dispersed in phosphate-buffered saline (PBS) solution are used as a reagent. For assaying tau protein, the antibody against tau protein is immobilized on these magnetic nanoparticles. Hereafter, such a reagent is referred to as the tau reagent. The ac magnetic susceptibility of the tau reagent is measured using a superconducting quantum interference device (SQUID) ac magnetic susceptometer^39^. Once the magnetic nanoparticles associate with the target molecules, the ac magnetic susceptibility signal is reduced. The reduction in the ac magnetic susceptibility of the reagent, referred to as the IMR signal, is a function of the concentration of target molecules. In previously published papers^37, 39^, significant IMR signals can be observed with a solution of 0.1 pg/ml tau protein. Although IMR sensitivity is ultra-high and it is a promising approach to precisely assay tau protein in human plasma, detailed examinations of the analytic performance of the tau reagent with IMR are absent from the literature. In this work, various experimental parameters, such as the Hook effect, limit of detection, assay linearity, dilution recovery range, assay reproducibility, reagent stability, interference, and spiked recovery, are investigated for the use of the tau reagent with IMR. Additionally, the IMR tau assay is applied to not only AD and healthy controls but also to patients with PD, FTD, and vascular dementia (VD) to explore the differences in plasma tau protein concentrations.

## Methods

### Constitution of the tau reagent

The tau reagent (MF-TAU-0060, MagQu) is a PBS solution that contains magnetic nanoparticles with a monoclonal antibody (T9450, Sigma) against human tau protein immobilized on their surfaces. The material of magnetic nanoparticle is Fe_3_O_4_, which is coated with dextran. Antibodies are covalently bound to the dextran. The mean value of the hydrodynamic diameter of antibody-functionalized magnetic nanoparticles is approximately 55 nm, measured using dynamic light scattering (Nanotrac 150, Microtrac). The concentration of the tau reagent is 8 mg Fe/ml.

### Measurement of IMR signal

For a given sample, 80 μl of the tau reagent was mixed with a 40-μl room temperature sample. The reduction in the ac magnetic susceptibility (i.e., the IMR signal) of the reagent after being mixed with a sample is measured using a SQUID-based ac magnetic susceptometer (XacPro-S, MagQu). To clarify the analytic performance of the tau reagent with IMR, several characterizations such as Hook effect, detection limit, assay linearity, dilution recovery range, assay reproducibility, reagent stability, interference test, and spike recovery, were investigated. The investigations were performed according to the global standardizations described by the Clinical & Laboratory Standards Institute (CLSI). The document numbers of the guidelines are EP5- A3, EP7-A2, EP17-A2, and C28-A2. Thus, the analytic performance of the tau reagent with IMR obtained by following the guidelines can be globally applied to clinical chemistry and laboratory medicine. The preparation of samples used for characterizing each type of analytic performance of the tau reagent is described in the Results and Discussion section.

### Recruitment of subjects

Subjects were recruited at National Taiwan University Hospital, Taiwan. All study subjects or their primary caregivers provided informed consent prior to participation in this investigation, and the study was approved by the ethics committee and the institute review board of National Taiwan University Hospital (Nos 201103059RB, 201301036RIND and 201406125DSC). All experiments were performed in accordance with relevant guidelines and regulations.

The exclusion and inclusion criteria for healthy controls and patients with MCI due to AD, AD, PD, FTD, or VD are listed in Table 1. The PD patients were at the stage of either PD with normal cognition, PD MCI or PD dementia. The demographic information of subjects is listed in Table 2.

**Table 1:** Exclusion and inclusion criteria for recruiting healthy controls and subjects with MCI due to AD, AD, PD, FTD, or VD in this study.

Group	Inclusion criteria	Exclusion criteria
Healthy controls	1. Education: at least primary school2. Age >50 years3. Body weight ≥40 kg4. CDR* = 05. MMSE^++^ ≥ 26	1. Subjects with cranial metallic implants, cardiac pacemakers or claustrophobia.2. Previous diagnosis of MCI or dementia3. Significant history of depression4. Geriatric Depression Scale >8
MCI due to AD	1. Subjects must meet the 2011 NIA-AA diagnostic guidelines for MCI due to AD based on memory impairment tested by WEMS-III^+^ and the score of any subtest below the 4th percentile and must be maintaining normal activities of daily living.2. Subjects must have MMSE scores between 24 and 28 and CDR = 0.5.	1. Subjects with cranial metallic implants, cardiac pacemakers or claustrophobia.2. Significant history of depression3. Geriatric Depression Scale >8
AD	1. Subjects must meet the 2011 NIA-AA diagnostic guidelines for probable AD dementia.2. Subjects must have MMSE scores between 10 and 22 and CDR = 0.5 or 1.	
FTD	1. Subjects must meet the diagnostic guideline for frontotemporal lobe degeneration (mainly primary progressive aphasia)^40^2. CDR = 0.5 or 1	
PD	1. Subjects must have symptoms of bradykinesia and at least one of the following: muscular rigidity, rest tremor (4–6 Hz), or postural instability unrelated to primary visual, cerebellar, vestibular or proprioceptive dysfunction.2. Three or more of the following symptoms: unilateral onset, rest tremor present, progressive disorder, persistent asymmetry affecting the side of onset most, excellent response to levodopa, severe levodopa-induced chorea, levodopa response for over 5 years, and clinical course of over 10 years.3. MOCA^#^ score greater than 26 for PD4. MOCA score less than 21 for PD with dementia	1. Significant history of depression2. History of repeated strokes with stepwise progression, repeated head injury, antipsychotic or dopamine-depleting drugs, definite encephalitis and/or oculogyric crises on no drug treatment, negative response to large doses of levodopa (if malabsorption excluded), strictly unilateral features after 3 years, other neurological features (supranuclear gaze palsy, cerebellar signs, early severe autonomic involvement, Babinski sign, early severe dementia with disturbances of language, memory or praxis), exposure to a known neurotoxin, or presence of cerebral tumor or communicating hydrocephalus on neuroimaging.
VD	1. Subjects must have stroke history with neuro-image confirmation.2. Subjects must have CDR > 0.5	1. Subjects with conscious disturbance or moderate to severe aphasia2. Significant history of depression3. Accompanied by other neurodegenerative diseases

*CDR: clinical dementia ranking.

^++^MMSE: mini-mental state examination.

^#^MOCA: Montreal cognitive assessment.

^+^WEMS-III: Wechsler Memory Scale Version III.

**Table 2:** Demographic information of subjects enrolled in this study. The concentration of total tau protein, ϕ_tau-IMR_ was detected using IMR. The age and ϕ_tau-IMR_ are presented in the form of the mean value ± standard deviation. The standard deviation is attributed to the variations among subjects.

Group	Numbers	Age (years)	ϕ_tau-IMR_ (pg/ml)	ApoE*ε*4 positive^&^
Healthy controls	66	64.6 ± 8.6	13.37 ± 7.77	25.0%
MCI due to AD^+^	24	71.0 ± 10.3	33.33 ± 7.77	36.3%
AD^++^	29	72.2 ± 9.9	55.44 ± 22.45	52.9%
PD^$^	41	67.1 ± 13.5	26.20 ± 8.37	—
FTD^^^	26	62.1 ± 9.2	41.28 ± 20.13	—
VD^#^	29	78.3 ± 4.4	19.96 ± 9.95	—

^+^MCI due to AD: mild cognitive impairment due to Alzheimer’s disease.

^++^AD: Alzheimer’s disease.

^$^PD: Parkinson’s disease.

^^^FTD: frontal temporal dementia.

^#^VD: vascular dementia.

^&^The analysis of ApoE*ε*4 allele was performed only for healthy controls and patients with MCI due to AD and AD.

### Preparation of human plasma

Subjects were asked to provide a 10-ml non-fasting venous blood sample (K3 EDTA, lavender-top tube). Colleagues were blind to all samples in the laboratory. The blood samples were centrifuged (1500–2500 g for 15 minutes) within 1 hour of the draw, and the plasma was aliquoted into cryotubes and stored at −20 °C.

### Statistical analysis

A multivariate analysis of variance (MANOVA) with a Bonferroni correction was used to examine group differences. A receiver operating characteristics (ROC) analysis was computed to identify possible useful cutoff points.

## Results

### Hook effect on assay

**Figure 1:**
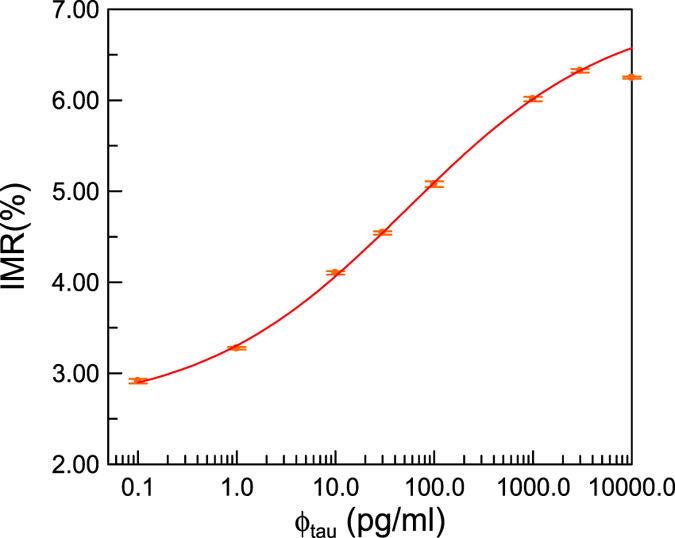
IMR signal as a function of the tau protein concentration in PBS solution. The error with each data point is attributed to the duplicated measurements of IMR signals. The solid line denotes the logistic function in Eq. (1). Hook effect occurs if the tau protein concentration is higher than 3,000 pg/ml.

Phosphate buffered saline (PBS) solutions spiked with various concentrations of tau protein, present as six isoforms (T7951; Sigma-Aldrich), were used as samples (referred to as Tau-PBS samples) for IMR measurements. The tau protein concentrations of Tau-PBS samples are 0.1, 1, 10, 30, 100, 1,000, 3,000, and 10,000 pg/ml. The IMR signals of these Tau-PBS samples are plotted in Fig. 1. The error bar associated with each data point in Fig. 1 is generated from duplicate measurements. It was found that the IMR signal, IMR(%), is larger for higher tau protein concentrations. However, the IMR signal for the 10,000-pg/ml Tau-PBS sample is lower than that for the 3,000-pg/ml Tau-PBS sample. The decrease in the IMR signal at tau protein concentrations higher than 3,000 pg/ml is a result of the Hook effect.

The IMR signals in Fig. 1 for tau protein concentrations from 0.1 pg/ml to 3,000 pg/ml are used for exploring the analytic relationship that follows the logistic function

1 $${\rm{IMR}}( \% )=[\frac{{\rm{A}}-{\rm{B}}}{1+{(\frac{{{\rm{\varphi }}}_{{\rm{tau}}}}{{{\rm{\varphi }}}_{{\rm{o}}}})}^{\gamma }}]\times 100 \% ,$$

where A, B, γ and ϕ_o_ are fitting parameters. By fitting the tau protein concentration-dependent IMR signals in Fig. 1 to Eq. (1), the parameters are found to be A = 2.59, B = 7.02, γ = 0.42 and ϕ_o_ = 53.58. The parameter ϕ_tau_ is the tau protein concentration. The fitted logistic function is depicted by the solid line in Fig. 1. Its coefficient of determination (R^2^) is 0.999.

### Assay detection limit

The global standards of the assay detection limit is described in CLSI EP17-A2, which provides guidance for evaluating the detection capacity of clinical laboratory measurement procedures (i.e., limit of blank and detection). According to CLSI EP17-A2, the limit of blank (LoB) should first be established, followed by finding the limit of detection (LoD). LoB is established as follows: the measurements are ordered according to their values, and the appropriate percentile (p) is estimated as the value of the observation with the rank value as determined below; in this case, p = 0.95:

2 $${\rm{LoB}}={\rm{Results}}\,{\rm{at}}\,{\rm{position}}[0.95\,\times \,{{\rm{N}}}_{{\rm{B}}}+0.5],$$

where N_B_ = 60 (N_B_ is the number of trials) in this case. Equation (2) becomes

3 $${\rm{LoB}}={\rm{Results}}\,{\rm{at}}\,{\rm{position}}\,57.5$$

This is a non-integer value. The distribution of 60 testing results exhibits a non-Gaussian distribution. Linear interpolation is carried out using the 57^th^ and 58^th^ ranked observations according to CLSI EP17-A2. The 60 measured concentrations for PBS samples that are not spiked with tau protein (i.e., blank samples) are ranked in Table 3. Using the 57^th^ and 58^th^ ranked observations for the linear interpolation, the 57.5^th^ (which denotes the mean of the measured concentrations of the 57^th^ and 58^th^ tests) observation indicates that the measured concentration is 0.01 pg/ml, which is the value of LoB for using the tau reagent with IMR to assay tau protein in PBS.

**Table 3:** Ranking list of the 60 measured tau protein concentrations for PBS samples not spiked with tau protein using the IMR tau reagent.

Rank	Measured concentration (pg/ml)	Rank	Measured concentration (pg/ml)
1	−0.12	31	−0.10
2	−0.12	32	−0.10
3	−0.12	33	−0.10
4	−0.12	34	−0.10
5	−0.12	35	−0.10
6	−0.11	36	−0.10
7	−0.11	37	−0.10
8	−0.11	38	−0.10
9	−0.11	39	−0.10
10	−0.11	40	−0.10
11	−0.11	41	−0.09
12	−0.11	42	−0.09
13	−0.11	43	−0.09
14	−0.11	44	−0.09
15	−0.11	45	−0.09
16	−0.11	46	−0.08
17	−0.11	47	−0.08
18	−0.11	48	−0.08
19	−0.11	49	−0.08
20	−0.11	50	−0.08
21	−0.11	51	−0.08
22	−0.11	52	−0.06
23	−0.10	53	−0.06
24	−0.10	54	−0.05
25	−0.10	55	−0.04
26	−0.10	56	−0.03
27	−0.10	57	0.00
28	−0.10	58	0.02
29	−0.10	59	0.02
30	−0.10	60	0.04

The limit of detection (LoD) is calculated via

4 $${\rm{LoD}}={\rm{LoB}}+1.645\,{{\rm{\sigma }}}_{{\rm{S}}},$$

where σ_S_ is the standard deviation of the measured tau protein concentrations of Tau-PBS samples at a given spiked tau protein concentration (e.g., 0.1 pg/ml in this work). The tau protein concentrations of 60 Tau-PBS samples were measured using the tau reagent with IMR. The measured concentration for each sample is listed in Table 4. The mean measurement of the 60 measured concentrations is 0.11 pg/ml. The σ_S_ of the 60 measured concentrations is 0.01 pg/ml. The LoD for assaying tau protein is 0.026 pg/ml using Eq. (4).

**Table 4:** List of the 60 measured tau protein concentrations for PBS samples spiked with 0.1 pg/ml tau protein using the IMR tau reagent.

Rank	Measured concentration (pg/ml)	Rank	Measured concentration (pg/ml)
1	0.09	31	0.12
2	0.10	32	0.12
3	0.10	33	0.12
4	0.10	34	0.12
5	0.10	35	0.12
6	0.10	36	0.12
7	0.10	37	0.12
8	0.10	38	0.12
9	0.10	39	0.12
10	0.10	40	0.12
11	0.11	41	0.12
12	0.11	42	0.12
13	0.11	43	0.12
14	0.11	44	0.12
15	0.11	45	0.12
16	0.11	46	0.12
17	0.11	47	0.12
18	0.11	48	0.12
19	0.11	49	0.12
20	0.11	50	0.12
21	0.11	51	0.12
22	0.11	52	0.12
23	0.11	53	0.12
24	0.11	54	0.12
25	0.11	55	0.12
26	0.11	56	0.12
27	0.11	57	0.13
28	0.11	58	0.13
29	0.11	59	0.13
30	0.12	60	0.13

### Assay linearity

**Figure 2:**
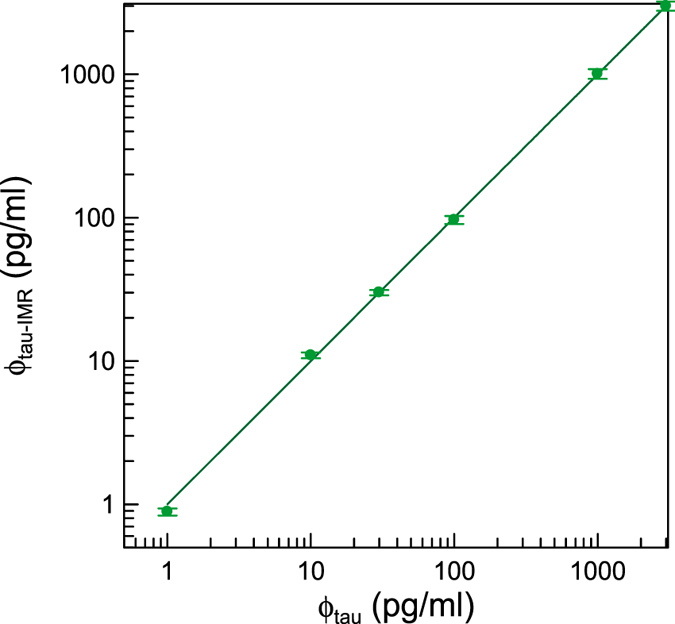
Measured tau protein concentration, ϕ_tau-IMR_, versus spiked tau protein concentration in PBS solution, ϕ_tau_. The error with each data point is attributed to the duplicated measurements of IMR signals. The solid line represents the proportionality between ϕ_tau-IMR_ and ϕ_tau_ with the slope of 1.00 for the ϕ_tau_ from 1 to 3,000 pg/ml.

The range of assay linearity was evaluated by comparing the tau protein concentration predicted by the IMR signal, ϕ_tau-IMR_, to the actual tau protein concentrations ϕ_tau_ of the Tau-PBS samples. Thus, the measured IMR signals of the Tau-PBS samples from 0.1 pg/ml to 3,000 pg/ml, as shown in Fig. 1, are converted to ϕ_tau-IMR_ via Eq. (1). The ϕ_tau-IMR_ versus ϕ_tau_ are plotted in Fig. 2. The relationship between ϕ_tau-IMR_ and ϕ_tau_ was found to be

5 $${{\rm{\varphi }}}_{\mathrm{tau}-\mathrm{IMR}}={\rm{1.00}}{{\rm{\varphi }}}_{{\rm{tau}}}$$

The coefficient of determination R^2^ is 0.999, and the correlation coefficient is 0.999. The acceptance criteria of the slope and correlation coefficient in the ϕ_tau-IMR_ − ϕ_tau_ curve are 0.9~1.1 and ≥0.95, respectively. The test results meet the acceptance criteria. Hence, the analytical measurement range for assaying tau protein using the tau reagent with IMR spans from 0.1 to 3,000 pg/ml.

### Dilution recovery range

One sample of PBS solution spiked with a known, purified Tau concentration (1018.57 pg/ml, measured with IMR) was diluted by factors of 5, 10, 20, 50 and 100 with PBS solution. The expected tau protein concentrations of these diluted samples were determined to be 203.57, 101.86, 50.93, 20.37, and 10.19, respectively, by dividing 1018.57 pg/ml by the dilution factors. The measured tau protein concentrations of these diluted samples were assayed using the tau reagent with IMR, as listed in Table 5. From the expected concentration and the measured concentration, the dilution recovery can be determined by

6 $${\rm{Dilution}}\,{\rm{recovery}}=\frac{{\rm{Measured}}\,{\rm{concentration}}}{{\rm{Expected}}\,{\rm{concentration}}}\times 100 \% $$

**Table 5:** Dilution factors, expected concentration, measured concentration, and dilution recovery for diluted samples used in the tests of dilution recovery range for assaying tau protein using the IMR tau reagent.

Dilution factor	Expected concentration (pg/ml)	Measured concentration (pg/ml)	Dilution recovery
5	203.71	221.35	108.7%
10	101.86	96.63	94.9%
20	50.93	52.26	102.6%
50	20.37	18.57	91.2%
100	10.19	8.46	83.0%

The dilution recoveries are listed in the right-most column of Table 3. The recoveries for tau protein solutions diluted at 1:5, 1:10, 1:20, and 1:50 ranged from 91.2% to 108.7%, which are within the acceptable dilution recovery range, i.e., from 90% to 110%. The recovery of the tau protein solution diluted by a factor of 100 is lower than 90%. This result implies that the sample used for IMR tau protein assay should not be diluted more than 100 times.

### Assay reproducibility

Reproducibility testing was conducted in accordance with the CCLS EP5-A3: Approved Guidelines for Evaluation of Precision Performance of Quantitative Measurement Methods. The Tau-PBS samples were measured in duplicate in one run. Two sequential measurements containing two duplicate measurements each are regarded as two runs. Two different and unknown tau protein concentrations were used for the tests. The measured tau protein concentrations ϕ_tau-IMR_ using the tau reagent with IMR are listed in Table 6 (Tau-PBS sample 1) and 7 (Tau-PBS sample 2). The mean concentrations of each pool are 9.91 pg/ml (Tau-PBS sample 1) and 95.46 pg/ml (Tau-PBS sample 2).

**Table 6:** Measured tau protein concentrations (listed in the columns of Result1 and Result2) in Tau-PBS sample 1 for the analysis of precision and reproducibility using the IMR tau reagent.

Day	Date	Run1				Day	Date	Run2				(Mean1−Mean2)^2^	Mean = (Mean1−Mean2)^2^
		Results1	Result2	Mean1 = (Result1 + Result2)^2^	(Result1−Result2)^2^			Results1	Result2	Mean2 = (Result1 + Result2)^2^	(Result1−Result2)^2^		
1	11/6/2015	10.34	10.86	10.60	0.26	2	11/6/2015	10.86	9.85	10.35	1.02	0.0608	10.48
3	11/12/2015	10.10	11.39	10.74	1.69	4	2/1/2016	8.07	10.10	9.08	4.08	2.7570	9.91
5	2/1/2016	8.07	10.10	9.08	4.08	6	2/12/2016	8.71	10.86	9.78	4.64	0.4864	9.54
7	2/14/2016	11.12	10.34	10.73	0.61	8	2/15/2016	8.49	10.60	9.54	4.44	1.4149	10.14
9	2/18/2016	10.86	10.34	10.60	0.26	10	2/19/2016	8.71	10.60	9.65	3.58	0.9012	10.13
11	2/20/2016	9.15	8.92	9.04	0.05	12	2/23/2016	11.67	9.85	10.76	3.31	2.9730	9.90
13	2/24/2016	11.39	10.34	10.87	1.10	14	2/25/2016	9.61	8.49	9.05	1.26	3.3060	9.96
15	2/26/2016	11.12	10.10	10.61	1.06	16	2/27/2016	10.86	9.85	10.35	1.02	0.0649	10.48
17	2/28/2016	8.92	10.60	9.76	2.80	18	2/29/2016	9.61	8.49	9.05	1.26	0.5048	9.42
19	7/19/2016	7.11	11.67	9.39	20.83	20	7/19/2016	9.85	8.28	9.07	2.47	0.1050	9.23
Sum					32.76						27.08	12.5740	99.19

Following the statistical method described in the CCLS EP5-A3: Approved Guidelines for Evaluation of Precision Performance of Quantitative Measurement Methods, an analysis of the results in Tables 6 and 7 yields the within-lab precision and standard deviations of repeatability, which are shown in Table 8. The imprecision (%CV) of assaying tau protein using the tau reagent with IMR is less than 15%.

**Table 7:** Measured tau protein concentrations (listed in the columns of Result1 and Result2) in Tau-PBS sample 2 for the analysis of precision and reproducibility using the IMR tau reagent.

Day	Date	Run1				Day	Date	Run2				(Mean1−Mean2)^2^	Mean = (Mean1−Mean2)^2^
		Results1	Result2	Mean1 = (Result1 + Result2)^2^	(Result1−Result2)^2^			Results1	Result2	Mean2 = (Result1 + Result2)^2^	(Result1−Result2)^2^		
1	12/27/2015	84.67	98.75	91.71	198.15	2	12/28/2015	100.95	86.55	93.75	207.49	4.1583	92.73
3	12/30/2015	84.67	96.60	90.64	142.18	4	12/31/2016	107.88	86.55	97.21	455.00	43.2783	93.93
5	1/6/2016	90.43	86.55	88.49	15.07	6	1/7/2016	90.43	107.88	99.16	304.47	113.7500	93.82
7	1/8/2016	98.75	100.95	99.85	4.85	8	1/13/2016	79.30	103.21	91.25	571.76	73.9465	95.55
9	1/14/2016	96.60	100.95	98.78	18.97	10	1/18/2016	90.43	88.47	89.45	3.85	86.9840	94.11
11	1/21/2016	98.75	100.95	99.85	4.85	12	3/3/2016	86.55	88.47	87.51	3.68	152.3640	93.68
13	4/1/2016	96.60	100.95	98.78	18.97	14	2/25/2016	88.47	94.50	91.48	36.32	53.2109	95.13
15	4/15/2016	103.21	100.95	102.08	5.09	16	2/27/2016	98.75	86.55	92.65	148.87	88.9489	97.37
17	4/17/2016	112.78	100.95	106.87	139.77	18	2/29/2016	81.05	100.95	91.00	396.20	251.6520	98.93
19	4/21/2016	82.84	100.95	91.90	328.07	20	7/19/2016	100.95	106.87	106.87	139.77	224.0283	99.38
Sum					875.98						2267.42	1092.3212

**Table 8:** Standard deviations of repeatability and within-lab precision for assaying tau protein concentrations in PBS using the tau reagent with IMR. The used samples show the mean measured total tau protein concentrations of 9.91 pg/ml and 95.46 pg/ml. The coefficient of variation is the ratio of the standard deviation to the mean of measured total tau protein concentrations.

Material	Mean of measured total tau protein concentrations	Standard deviation (Coefficient of variation)	
		Repeatability	Within-Lab
Tau-PBS sample 1	9.91 pg/ml	1.22 pg/ml (12.3%)	1.12 pg/ml (11.3%)
Tau-PBS sample2	95.46 pg/ml	8.86 pg/ml (9.3%)	8.49 pg/ml (8.9%)

### Reagent stability

A Tau-PBS sample is used to test reagent stability. The tau reagent was stored at 2–8 °C during the test. The variation in the measured tau protein concentration of the Tau-PBS sample on different days is listed in Table 9. The standard deviation of each measured concentration was determined from duplicate measurements. The measured concentration on Day 0 (week 0) is used as a reference, and the *p* value of the measured concentrations in other weeks are calculated and are listed in the right-most column in Table 9. All *p* values are higher than 0.05. This result implies that there is no significant variation among these measured concentrations. The % drift of the measured tau protein concentration, in comparison with Day 0, at each time point was within the range −10% to 10%. Therefore, the data demonstrate a 166-day stability period for the tau reagent when stored at 2–8 °C.

**Table 9:** Variation in the measured tau protein concentration in the Tau-PBS sample measured on different days by using the IMR tau reagent.

Storage period (Day)	Measured concentration (pg/ml)	*p*	% Drift
0	91.71 ± 9.95	−	−
50	94.89 ± 9.73	0.39	3.46
77	86.56 ± 0.31	0.27	−5.62
106	87.11 ± 0.69	0.29	−5.01
133	93.83 ± 11.70	0.43	2.30
166	87.67 ± 0.12	0.31	−4.41

### Interference test

Human plasma may contain materials that can interfere with these measurements, such as hemoglobin, bilirubin or intralipid (associated with diseases such as hemolysis, jaundice or hypertriglyceridemia). Other biomaterials naturally exist in plasma, such as uric acid, rheumatoid factor or albumin, which also interfere. Drugs or chemicals in medicine used to treat inflammatory diseases, viral and bacterial infections, cancers and cardiovascular disease may also interfere. Each of the natural biomaterials and drugs or chemicals tabulated in Table 10 were added to individual PBS solutions, which also contained 100 pg/ml tau protein. Their concentrations are listed in Table 8. Note that the concentrations of the interfering materials used in this study are much greater than ordinary levels. For example, the level of hemoglobin in the blood of a patient with hemolysis is approximately 500 μg/ml. The concentration of hemoglobin used in Sample No. 2 is 1000 μg/ml. The measured tau protein concentrations for these 100-pg/ml Tau-PBS solutions are listed in Table 10. The measured tau protein concentration for the PBS solution (Sample No. 1) with only 100-pg/ml tau protein is used as a reference. All the measured tau protein concentrations for the other PBS samples (Sample Nos 2–15) with both 100-pg/ml tau protein and the interfering materials are compared with the reference tau protein concentration (Sample No. 1). The Mean % Recovery is determined by the ratio of the measured tau protein concentration of a sample to that of the reference sample (No. 1). Acceptable Mean % Recovery values range from 90.0% to 110.0%. The results showed that the Mean % Recovery of these tests ranges from 91.8 to 108.9, as shown in Table 8. This finding indicates that the biomolecules, drugs and chemicals listed in Table 10 do not interfere with the assay for tau protein using the tau reagent with IMR.

**Table 10:** Materials and their concentrations used for interference tests for tau protein assay by utilizing the tau reagent with IMR. The concentration of tau protein in each sample is 100 pg/ml. The matrix is PBS solution. The detected tau protein concentrations of each sample are listed. Using the tau protein concentration of the pure Tau-PBS sample (sample No. 1) as a reference, the Mean % Recovery values of the tau protein concentration for other samples are calculated and listed in the right-most column.

Sample No.	Interfering material	Concentration	Measured tau protein concentration (pg/ml)	Mean % Recovery
1	None	—	99.85	—
2	Hemoglobin	10000 μg/ml	99.21	99.4%
3	Conjugated bilirubin	600 μg/ml	91.67	91.8%
4	Intra lipid	30000 μg/ml	108.51	108.7%
5	Uric acid	200 μg/ml	91.67	91.8%
6	Rheumatoid factor	500 IU/ml	93.48	93.6%
7	Albumin	60000 μg/ml	95.80	95.9%
8	Acetylsalicylic acid	500 μg/ml	106.13	106.3%
9	Ascorbic acid	300 μg/ml	107.31	107.5%
10	Ampicillin sodium	1000 μg/ml	107.43	107.6%
11	Quetiapine Fumarate	100 ng/ml	107.61	107.8%
12	Galantamine hydrobromide	90 ng/ml	108.51	108.7%
13	Rivastigmine hydrogen tartrate	100 ng/ml	107.25	107.4%
14	Donepezil Hydrochloride	1000 ng/ml	100.45	100.6%
15	Memantine Hydrochloride	150 ng/ml	108.75	108.9%

### Spiked recovery

The tau protein concentration of a human plasma sample (No. PRA in Table 11) was determined via IMR assay to be 21.54 pg/ml. The other human plasma sample spiked with tau protein (No. PRF) was assayed using the tau reagent with IMR and found to be 1589.06 pg/ml. Sample PRF was spiked into sample PRA at various volume ratios, as listed in Table 9, to obtain tau plasma samples of various tau protein concentrations. The expected concentrations of various spiked tau plasma samples are listed in Table 11. The measured tau protein concentrations of these spiked human plasma samples were determined using the tau reagent with IMR, as listed in Table 11. The spiked recovery was calculated as the ratio of the measured concentration to the expected concentration. As shown in the right-most column in Table 11, the spiked recovery ranges from 90% to 110% with a mean of 96.8% for tau plasma samples using the tau reagent with IMR.

**Table 11:** Measured tau protein concentration using the IMR reagent and spiked recovery rate for spiked tau plasma samples.

Plasma sample No.	Volume ratio (PRA:PRF)	Original concentration (pg/ml)	Expected concentration (pg/ml)	Measured concentration (pg/ml)	Spiked recovery rate (%)
PRA	—	21.54	—	—	—
PRB	95%:5%	—	99.91	99.47	99.6
PRC	75%:25%	—	413.42	418.91	101.3
PRD	50%:50%	—	805.30	737.09	91.5
PRE	25%:75%	—	1197.18	1132.32	94.6
PRF	—	1589.06	—	—	—

### Plasma tau protein concentrations in dementia

**Figure 3:**
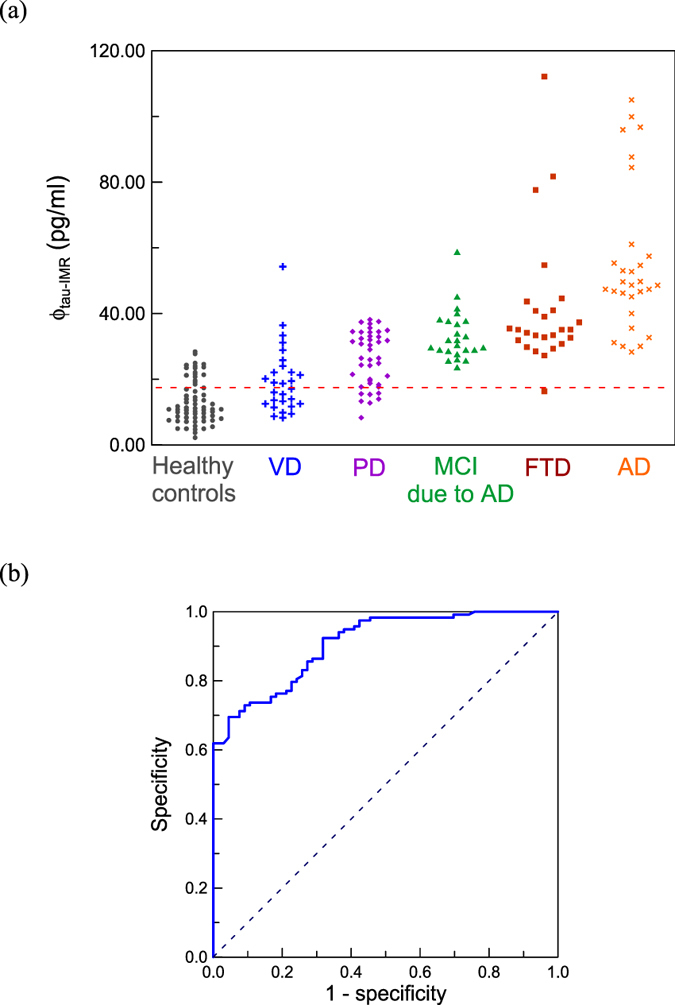
(**a**) Measured tau protein concentrations, ϕ_tau-IMR_, in human plasma for healthy controls and various types of dementia, including VD (), PD (), MCI due to AD (), FTD (), and AD (). (**b**) ROC curve for discriminating between healthy controls and dementia subjects in terms of plasma tau protein concentration. The cutoff value of plasma tau protein concentration to discriminate healthy controls from dementia subjects was 17.43 pg/ml, as plotted with the dashed line in (**a**). The corresponding clinical sensitivity and specificity are 0.856 and 0.742, respectively. The area under the ROC curve is 0.907.

The tau reagent with IMR was used to assay tau protein, ϕ_tau-IMR_, in plasma for healthy human controls and subjects with various types of dementia. The results are plotted in Fig. 3(a). Each data point in Fig. 3(a) denotes the tau protein concentration of a subject. The average values and the standard deviations of the measured tau protein concentrations, ϕ_tau-IMR_, for every group are listed in Table 2. The healthy controls exhibit the lowest level of plasma tau protein, whereas the AD patients show the highest level of plasma tau protein.

The detected concentrations of plasma tau protein shown in Fig. 3 range from 10 to 100 pg/ml, which is higher than that reported in other studies such as ref. 33 (1–10 pg/ml). The difference might be attributed from the assay technologies. The technology, so-called single molecule assay (SIMOA), was used to assay plasma tau protein in ref. 27. The SIMOA utilizes magnetic nanoparticles for the purification of tau protein molecules (or to concentrate tau protein molecules). This process usually causes loss of tau protein molecules. IMR is a direct measurement of plasma tau protein molecules. Hence, the levels of plasma tau protein molecules detected with SIOMA would be lower than that of IMR.

## Discussion

According to the results shown in Fig. 3, an average tau protein concentration ϕ_tau-IMR_ of (13.37 ± 7.77) pg/ml in the plasma was found in healthy controls (n = 66). The ϕ_tau-IMR_ in plasma of subjects suffering from VD (n = 29) was measured as (19.96 ± 9.95) pg/ml, which is slightly higher than that of healthy controls (*p* < 0.05). The results are consistent with the reported observations for the elevation of tau protein concentrations in the CSF of VD patients^41–43^.

The ϕ_tau-IMR_ was (26.20 ± 8.37) pg/ml for PD patients (n = 41), and this value is significantly larger than that for VD (*p* < 0.05). This result might imply that the neurofibrillary tangle formation is more common in PD than in VD. Remarkably, a clear difference in the plasma tau protein concentration was observed between healthy controls and PD patients (*p* < 0.001). Hence, PD may be related to tauopathy.

For the plasma tau protein concentration in patients with MCI due to AD (n = 24), the ϕ_tau-IMR_ values are (33.33 ± 7.77) pg/ml. The *p* value in ϕ_tau-IMR_ between MCI due to AD and PD is smaller than 0.001, indicating a significant difference in ϕ_tau-IMR_ between these two types of dementia. A significant difference in ϕ_tau-IMR_ was also found between FTD and MCI due to AD (*p* < 0.001). The FTD patients (n = 26) had a plasma tau protein concentration of (41.28 ± 20.13) pg/ml. The ϕ_tau-IMR_ values of AD patients (n = 29) showed the highest level, (55.44 ± 22.45) pg/ml, resulting in a *p* value smaller than 0.05 compared with the ϕ_tau-IMR_ of FTD. It should be noted that AD and MCI due to AD are highly related to tauopathy (i.e., neurofibrillary tangle formation of neurons). The fact that FTD has a tau protein level between those of MCI due to AD and AD suggests that the neurofibrillary tangle formation plays a role in causing FTD. This fact is consistent with observations of neurofibrillary tangles in the brain biopsies of FTD patients.

By combining all patients in the category of dementia subjects, an analysis of the receiver operating characteristic (ROC) curve was conducted to discriminate between healthy controls and dementia subjects in terms of plasma tau protein concentration; the results are shown in Fig. 3(b). The cutoff value for the plasma tau protein concentration is 17.43 pg/ml, as plotted with the dashed line in Fig. 3(a). The corresponding clinical sensitivity and specificity are 0.856 and 0.742, respectively. The area under the curve is 0.907. These results support the feasibility of screening for dementia in VD, PD, MCI due to AD or AD by assaying plasma tau protein. The clinical sensitivity of screening each type of dementia, using 17.43 pg/ml as a cutoff value for the plasma tau protein concentration, was calculated and is listed in Table 12. The clinical sensitivity between VD and healthy controls is 0.517. The clinical sensitivity between healthy controls and either that of PD, MCI due to AD, FTD or AD is higher than 80%. Therefore, plasma tau protein is a promising biomarker for screening for VD, PD, MCI due to AD, FTD and AD. Note, the clinical sensitivity of discriminating VD from healthy controls using plasma tau protein levels is 0.517. This finding means that the plasma tau protein concentrations of VD mostly overlap with those of healthy controls. Although some reports showed an elevation of CSF tau protein concentration in VD compared to healthy controls^41–43^, other published papers demonstrated no significant increase in CSF tau protein levels in VD^44, 45^. Furthermore, pathological evidence was given to reveal insufficient neurofibrillary tangles in temporal and frontal cortices in VD^46–48^. The inconsistency among studies on tauopathy in VD might be a result of the severity of cerebrovascular disease, age, or the acute or chronic phase of brain ischemia^41, 44, 49^. More effects are needed to clarify the role of each factor in VD.

**Table 12:** Clinical sensitivity for discriminating between each type of dementia and healthy controls using 17.43 pg/ml of plasma tau protein as a cutoff value.

	VD	PD	MCI due to AD	FTD	AD
Healthy controls	0.517	0.829	1.000	0.961	1.000

## Conclusion

The tau reagent consisting of magnetic nanoparticles functionalized with antibodies and dispersed in PBS solution has been developed for IMR assay. The measurement range for the tau protein using the tau reagent with IMR ranges from 0.1 to 3,000 pg/ml, and the measurement imprecision is less than 15%. Additionally, no interference was observed in this assay in the IMR results. Thus, the tau reagent with IMR method has high sensitivity and high specificity. By applying IMR to assaying tau protein in human plasma, the tau protein level for healthy controls was found to be approximately 13 pg/ml, which is relatively lower than that for VD, PD, MCI due to AD, FTD, and AD. Furthermore, the plasma tau protein level increases in the sequence of VD, PD, MCI due to AD, FTD, and AD. These results demonstrate the possibility of assaying plasma tau protein for screening for neurodegenerative diseases.
